# Epigenetic analysis confirms no accelerated brain aging in schizophrenia

**DOI:** 10.1038/s41537-017-0026-4

**Published:** 2017-09-04

**Authors:** Joanne Voisey, Bruce R. Lawford, C. Phillip Morris, Leesa F. Wockner, Ernest P. Noble, Ross McD Young, Divya Mehta

**Affiliations:** 1 School of Biomedical Sciences, Faculty of Health, Institute of Health and Biomedical Innovation, Queensland University of Technology, Brisbane, QLD Australia; 2 School of Psychology and Counselling, Faculty of Health, Institute of Health and Biomedical Innovation, Queensland University of Technology, Brisbane, QLD Australia; 3 Queensland Institute of Medical Research, Brisbane, QLD Australia; 4 Department of Psychiatry and Biobehavioral Sciences, University of California, Los Angeles, CA USA; 5 Faculty of Health, Institute of Health and Biomedical Innovation, Queensland University of Technology, Brisbane, QLD Australia

## Abstract

Epigenetic aging is associated with several biological mechanisms and diseases. We assessed two brain data sets, one small (*n* = 48) and one large (*n* = 392), to test epigenetic aging in schizophrenia. DNA methylation age from frontal cortex was significantly correlated with chronological age but no significant differences in DNA methylation age acceleration between schizophrenia cases and controls were observed in both data sets. Our results were consistent with a previous study investigating schizophrenia and epigenetic aging in superior temporal gyrus. Future studies targeting different brain regions and defined cell types are warranted to further investigate accelerated brain aging in schizophrenia.

## Introduction

Several lines of evidence have pointed towards the hypothesis of accelerated brain aging in schizophrenia.^1, 2^ In recent years, DNA methylation of 353 CpG sites (epigenetic clock) have been used to accurately estimate the biological age of human tissues and cell types.^3^ Studies have found epigenetic aging to be associated with Alzheimer’s disease,^4^ obesity^5^ and cancer.^6^ Only one study has investigated epigenetic aging in schizophrenia.^7^ Using postmortem brain samples from the superior temporal gyrus (*n* = 44), no acceleration of brain aging in schizophrenia was identified.

The aim of this study was to test the hypothesis of accelerated aging in schizophrenia by assessing epigenetic age in brain tissue of individuals with schizophrenia. Using a different brain region, the frontal cortex, we investigated a total of 440 samples including data from our previous study^8^ (*n* = 48) as well as an independent published data^9^ (*n* = 392).

## Methods

Genome-wide DNA methylation analysis was generated from post-mortem human brain tissue (frontal cortex) from 24 individuals with schizophrenia and 24 unaffected controls. DNA methylation was assessed using the Illumina Infinium HumanMethylation450 Bead Chip, details are described in a previous study.^8^ DNA methylation-based age prediction was performed using the statistical pipeline developed by Horvath.^3^ The raw data were normalized using BMIQ normalization method. To measure epigenetic age acceleration effects, we regressed DNAm age on chronological age to obtain the DNAm age acceleration residuals. Hence, age acceleration would denote individuals who appear to be older than their chronological age. Next, we regressed DNAm age acceleration residuals against the group status, adjusting for gender, brain post-mortem interval and cause of death. Similar methods were used for the independent published replication data set as in the original study adjusting for gender, four principal components and ethnicity.^9^ From the published data set, we limited our analysis to adult samples that passed the QC including 217 controls and 175 Schizophrenia, comprising a total of 392 samples (Supplementary Table 1).

Ethics approval for the project was obtained from the Human Research Ethics Committee of the Queensland University of Technology.

### Data availability

Discovery data set https://www.ncbi.nlm.nih.gov/geo/query/acc.cgi?acc=GSE61107

Replication data set https://www.ncbi.nlm.nih.gov/geo/query/acc.cgi?acc=GSE74193

## Results

**Fig. 1:**
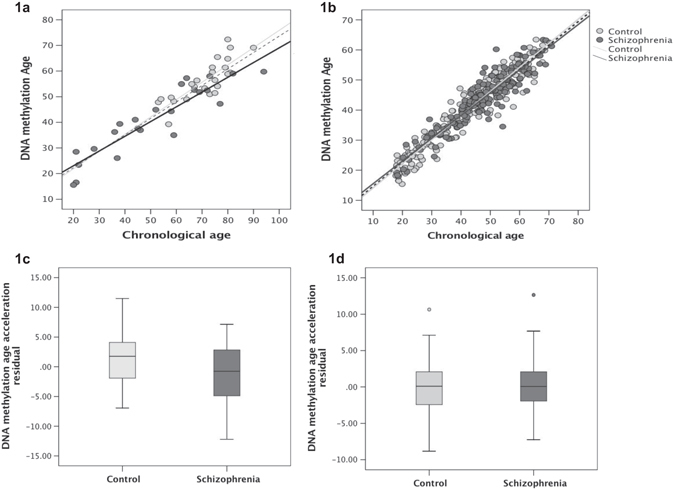
No evidence of accelerated aging in brain tissue in schizophrenia—**a** and **b** indicate the significant correlation between the chronological and epigenetic age in our sample (**a**) (*r* = 0.92, *p*-value = 4.626603e-20) and the replication sample (**b**) (*r* = 0.9454137 *p*-value 5.237090e-192). **c** and **d** indicate no differences in DNA methylation age acceleration in our sample **c** (*p*-value = 0.095) and the replication sample (**d**) (*p*-value = 0.702)

Demographics of the samples are shown in Table 1. There was a significant difference in PMI and age across the samples, but no significant differences in DNA methylation age acceleration. All results are indicated in Fig. 1. In the overall sample (*n* = 48), DNA methylation age was significantly correlated with chronological age (*r* = 0.92, *p*-value = 4.626603e-20). Similar results were observed in schizophrenia cases (*r* = 0.92 and *p*-value = 8.62e-10) and in controls (*r* = 0.82 and *p*-value = 7.2e-07).

**Table 1:** Demographics of the 48 samples used in the study

	Total (*n* = 48)	Scz (*n* = 24)	Controls (*n* = 24)	*p*-value
	Mean [SE] or *N* [%]			
Age	61.66 [2.80]	52 [4.5]	71.3 [2.0]	0.000192
PMI	18.84 [1.32]	24 [2.17]	14.1 [0.67]	0.000075
Gender—males [%]	35 [73%]	16 [67%]	19 [79%]	0.451
Cause of dealth—suicide	5 [10%]	5 [21%]	0 [0%]	0.018
Age acceleration residual	1.33e-15 [0.80]	1.30 [0.93]	−1.35 [1.17]	0.082

When comparing the groups, no significant differences in DNAm age acceleration was seen between the schizophrenia cases and controls (*p*-value = 0.08), after adjusting for covariates. No differences in DNAm age acceleration were observed when stratifying by gender or by taking the mean or median age of the sample (*p*-value > 0.05). The average delta age (DNAm-age—chronological age) in the schizophrenia cases was −10.4 and that in the controls was −14.8 years, indicating that on average, the controls had 4 years increased age acceleration than the schizophrenia cases.

We replicated our findings using 392 samples (217 controls and 175 Schizophrenia) from another study which investigated DNA methylation in prefrontal cortex.^9^ In the overall replication sample, DNA methylation age was significantly correlated with chronological age (*r* = 0.9454137, *p*-value = 5.237090e-192). Similar results were observed in schizophrenia cases (*r* = 0.9292716, *p*-value = 9.775933e-77) and in controls (*r* = 0.9517476, *p*-value = 2.853058e-112). As in the discovery sample, no significant differences in DNAm age acceleration was seen between the schizophrenia cases and controls (*p*-value = 0.916) also when stratifying by gender or by taking the mean or median age of the sample (*p*-value > 0.05). The average delta age (DNAm-age—chronological age) in the in the controls was −2.3 years and that schizophrenia cases was −3.7.

## Conclusions

This is the first study in frontal cortex and only the second study using brain tissue to investigate the relationship between epigenetic aging and schizophrenia. Although schizophrenia is associated with age-related physiological factors as well as significant decrease in average life span,^10^ we were unable to confirm accelerated aging in our study. These results suggest that brain volume loss observed in schizophrenia might be explained by pathological processes other than accelerated aging. Our results are limited since this is a cross sectional study and we were unable to assess progressive age acceleration that might occur in the brain of individuals with schizophrenia. Also, it is likely that there might be other confounding factors that might influence our results.

In conclusion, our findings in frontal cortex of individuals with schizophrenia are consistent with those from McKinney et al.^7^ who reported lack of evidence for accelerated epigenetic aging in schizophrenia superior temporal gyrus region of the brain. Nevertheless, we cannot rule out the possibility of other aging mechanisms that might be independent of epigenetic aging in schizophrenia brain and/or accelerated epigenetic aging that might be present in other tissues. Future studies aimed at testing the accelerated aging hypothesis in other brain regions as well as defined cell types will further uncover the hypothesis of accelerated brain aging in schizophrenia.

## Electronic supplementary material

Demographics Table
